# Telenursing in the sexual function of women with breast cancer: A study protocol

**DOI:** 10.1097/MD.0000000000031449

**Published:** 2022-11-25

**Authors:** Iarlla Silva Ferreira, Ana Fátima Carvalho Fernandes, Régia Christina Moura Barbosa Castro, Andrea Rodrigues Bezerra, Romel Jonathan Velasco Yanez

**Affiliations:** a Department of Nursing at the Federal University of Ceará, Fortaleza-CE, Brazil.

**Keywords:** breast neoplasms, clinical trial as topic, sexuality, telenursing

## Abstract

Most women with breast cancer have sexual function problems related to cancer diagnosis and treatment. These problems harm the quality of life and relationships. However, most patients do not receive care or guidance regarding sexuality from healthcare providers. This study aimed to test the effect of telenursing counseling on sexual functioning in women undergoing breast cancer treatment.

**Methods:**

This randomized clinical trial was conducted at 2 reference hospitals for cancer treatment. One hundred eight women with stage I–IV breast cancer undergoing treatment (surgery, chemotherapy, radiotherapy or hormone therapy) with a partner will be included in this study. The study was authorized by the Ethics Committee of the Federal University of Ceará (Opinion number: 46,13,609) and the Maternity School Assis Chateaubriand (Opinion number: 47,42,687). Patients will be allocated to the following groups: the control group, which will not receive an active intervention, and the intervention group, which will receive 3 telenursing counseling sessions for 6 weeks. The levels of sexual function in these patients before the intervention, soon after the intervention and at 12 weeks were compared and analyzed. All data will be collected and analyzed by the JASP program.

**Results:**

Differences in levels of sexual function among women allocated to the control and intervention groups in the analyzed periods.

**Conclusion:**

This evidence-based nursing care strategy can be used to improve the sexual function of breast cancer patients and consequently their quality of life and marital relationship.

## 1. Introduction

There is a consensus in the literature that most breast cancer patients have sexual problems. These problems are due to the diagnosis or cancer treatment, including surgery, chemotherapy, radiotherapy, and hormonal therapy, which interfere directly or indirectly with sexual functioning, whether through psychological, social, or biological aspects.^[1,2]^

Changes in the sexual functioning of breast cancer patients start from treatment and may last for a long period. The younger the patient, the more severe the symptoms or sexual problems will be. These problems directly impact the quality of life of these patients and their relationships.^[3,4]^ One of the major problems surrounding this issue is the fact that many healthcare practitioners do not address the sexuality complaints of patients with breast cancer during their appointments. Therefore, most patients seek information from other sources, thus highlighting communication barriers in the professional-patient relationship and the maintenance of a taboo around sexuality and its relevance to women, especially in the context of breast cancer treatment.^[5,6]^

This problem may have been further aggravated by the pandemic context experienced in the last 2 years, especially due to the need to reduce personal interactions to minimize the risk of COVID-19 transmission. Implementing healthcare and health assessment tools that do not expose professionals or cancer patients to COVID-19 contributed to the increase in telehealth activities delivered via phone or video.^[7]^

Few studies have examined how educational interventions by phone impact the sexual function of women with breast cancer undergoing treatment,^[8–10]^ especially at the beginning of cancer treatment and led by nurses. The International Council of Nurses considers telenursing a service that enables the maintenance of effective communication with clients with chronic noncommunicable diseases, in addition to providing an effective intervention in the promotion and education for a healthy life.^[11]^

There is, therefore, a need to evaluate the impact of educational interventions performed through telenursing on the sexual functioning of women with breast cancer more deeply, aiming to guide and inform these patients about the sexual problems arising from cancer diagnosis and treatment and help them deal with these problems. This study aims to test the effect of telenursing counseling on the sexual functioning of women undergoing breast cancer treatment.

## 2. Materials and methods

### 2.1. Study design

This is a randomized clinical trial (RCT) with 2 parallel groups, with an allocation ratio of 1:1, with implementation between February 2022 and February 2023. This protocol was developed according to the guidelines of the Standard Protocol Items: Recommendations for Interventional Trials (SPIRIT)^[12]^ (Supplemental data, Supplemental Digital Content, http://links.lww.com/MD/xxx).

### 2.2. Study setting

The study will be conducted in 2 reference hospitals in the state of Ceará, Brazil. One is a university hospital linked to the Federal University of Ceará. The other is a reference center for diagnosing, treating, and monitoring cancer in Ceará.

### 2.3. Subjects

The study population will consist of women with breast cancer undergoing cancer treatment recruited using the following inclusion criteria: stage I, II, III or IV breast cancer; oncological treatment (surgery, chemotherapy, radiotherapy, or hormone therapy), as the literature indicates that all treatments interfere directly or indirectly in sexual functioning and that these effects may begin from treatment and remain for a long period;^[4,13]^ having at least one telephone number; and having a partner or spouse. The following subjects will be excluded: patients in treatment for sexual dysfunction or climacteric symptoms to reduce confounding variables in the assessment of the intervention’s outcome;^[14]^ treatment for another type of cancer; hearing impairment, as it makes communication by phone impossible; and having a medical diagnosis of any mental disorders since cognitive functioning is necessary to understand and fill in the data collection instruments.

The discontinuity criteria are patients’ withdrawal from participating in the research after the commencement of data collection, death during the study, phone change after follow-up, and not answering phone calls after 5 attempts on different dates and times. There will be no change in the participant’s allocation to minimize the risk of bias.

### 2.4. Sample size

G*power software, Version 3.1.9.6 (German University Heinrich-Heine Universitaet, Duesseldorf)^[15]^, was used to calculate the sample size by selecting “Test F” as the family of the test, considering that the analysis of variance (ANOVA) of repeated measures with intra- and intergroup interaction will be used to verify the null hypothesis.

The sample size estimation was made using an equation based on ANOVA, which took into account an effect of 0.085 (representing an effect size of at least 0.30 in the groups) and a power of 80% in the repeated measures design (2 groups, alpha = 0.05, nonsphericity correction = 0.5). Given the parameters above, the G*power software suggested 45 participants in each group (90 participants in total). Taking into account possible sample losses, a percentage of 20% was added, which totaled a sample of 108 participants.

This effect size was stipulated by a previous study, which identified an intermediate effect size (approximately 30%) for sexual functioning among the groups (control and intervention).^[16]^ The sphericity corresponds to one of the assumptions the data must meet so that ANOVA can be used, ranging from 0.5 to 1, with 0.5 being the most rigorous.^[15]^

### 2.5. Recruitment and data collection

The patients will be approached in person at the study locations by a team of 2 researchers who will contribute to the data collection process. These researchers received a training of approximately 8 hours on the objectives and methodological procedures of the study, where simulations involving the data collection instrument were also performed. These researchers are also members of a research group that conducts research on mastectomized women at the Nursing Department of the Federal University of Ceará. After formal consent, sociodemographic and clinical data will be collected from the medical records and through the application of an adapted questionnaire.^[17]^ The questionnaire analyzes the following variables: age, education, color, marital status, length of the relationship, the average number of sexual intercourse/month, occupational status, presence of sexual problems before the diagnosis of breast cancer, city of origin, family income, religion, children, and the number of residents at home. Moreover, the following clinical data will be collected: weight, height, body mass index, menstruation status, presence of comorbidities, risk factors for breast cancer, time of diagnosis, primary tumor, lymph node and metastasis staging, previous surgery, therapeutic intervention currently implemented, and therapeutic interventions previously performed (Table 1).

**Table 1:** Schedule of enrollment, interventions, and assessments.

Study period								
Timepoint	Enrolment	Allocation	Post-allocation					Close-out
	-t1	0	t1	t2	t3	t4	etc.	tx
ENROLMENT:								
Eligibility screen	X							
Informed consent	X							
*[List other procedures]*	X							
Allocation		X						
INTERVENTIONS:								
*[Counseling by telenursing]*								
*[Untreated]*								
ASSESSMENTS:								
*[List baseline variables]*	X							
*[List outcome variables]*	X					X		X
*[List other data variables]*						X		

**Figure 1.**
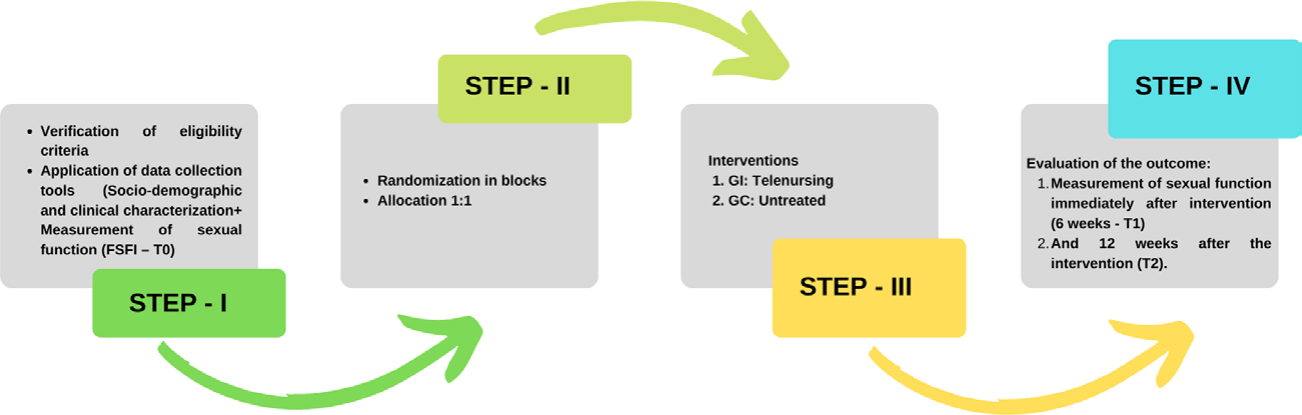
Flowchart of the data collection steps.

Sexual functioning, which corresponds to the main outcome, will be analyzed using the Female Sexual Function Index (FSFI), an instrument of a specific nature and multidimensional scope that evaluates female sexual function. The scale has a possible maximum of 36 and a minimum of 2, which is obtained by summing the weighted scores of each of the 6 domains (desire, subjective arousal, orgasm, satisfaction, and pain). Higher scores reflect a better degree of sexual function^[18]^ (Fig. 1).

### 2.6. Allocation

Eligible participants will be randomly allocated to the intervention group or control group, with an allocation ratio of 1:1, using a simple randomization technique.^[14]^ After completion of the baseline evaluations, eligible patients will be randomized by a member of the research group that is not linked to the study. This member will assign a random numbering to each participant and then carry out a draw from the participants who will compose each group. Then, the principal researcher will carry out the intervention after being informed about the allocation of subjects in the groups.

### 2.7. Interventions

The control group will not receive any intervention but may receive orientations about sexuality by healthcare professionals such as social workers, nurses, psychologists, or physicians (oncologists, mastologists, or gynecologists), eventually considering that none of the institutions where the study will be undertaken has specific protocols or routines aimed at sexual and reproductive health.

Three telenursing counseling sessions will be carried out over 6 weeks in the intervention group. This period was chosen for being considerate enough to address the issues planned and to establish a bond and a relationship of trust between the researchers and the participants. Most educational interventions aimed at the sexual functioning of women with breast cancer are carried out in a period of 3 to 24 weeks.^[8,16,19,20]^ The sessions will take place at intervals of at least 15 days, considered enough for contacting all participants but not so long that would cause forgetfulness of the information provided in each contact.^[21]^

The calls will be made at times and days arranged with the participants and the researcher and are estimated to last 30 to 40 minutes. The telephone counseling will be conducted exclusively by the principal researcher to minimize the risks of divergence in the approach. The principal researcher has worked as a nurse practitioner in the public health system for approximately seven years, has a specialization degree in public health, has a master’s degree in nursing, and has worked with sexual dysfunction in women with breast cancer for 4 years.

The content of the counseling sessions will be based on the guidelines provided by the American Cancer Society for managing female sexual problems related to cancer. The contents were organized into 3 main topics: Cancer, sex, and the female body; How can treatments interfere with sexual life? And: managing female sexual problems related to cancer.^[22]^ The counseling sessions will be based on the constructs of social-cognitive theory.

Telenursing uses technologies to provide nursing care and direct nursing practice.^[23]^ Moreover, since 2008, the International Council of Nurses has considered telenursing a service that provides nurses with the administration of care to patients living in rural or remote areas and enables them to maintain effective communication with clients who have noncommunicable chronic diseases, providing an effective strategy to promote self-care and a healthy lifestyle.^[22]^ The telephone intervention eliminates visual contact, providing anonymity during the interactions. Furthermore, telephone calls enable greater inclusion of patients who live in rural areas or locations that are difficult to reach and who are often not prone to in-person interventions. Finally, telephone calls are considered an economical intervention method.^[24]^

### 2.8. Relevant concomitant care permitted or prohibited during the trial

Concomitant treatments for sexual dysfunction or menopausal symptoms will not be allowed during participation in the study since the comparison between the control and intervention groups does not allow cointerventions (medications, therapies, or behaviors) to avoid interferences regarding the outcome.^[14]^

### 2.9. Outcome

The study’s primary outcome will be sexual functioning, which will be assessed using the FSFI. The FSFI is an instrument built and validated in English^[18]^ and currently has at least 20 translations in different languages. The initial validation of the FSFI obtained a Cronbach’s alpha > 0.90 and reliability coefficient in the high general retest for all domains that make up the scale (*R* = 0.79 to 0.86), demonstrating good construct validity with a statistically significant difference between the averages of groups with and without sexual desire disorder (*P* < .001). The data above support the psychometric reliability and validity of the FSFI. In 2007, the FSFI was translated and validated into Portuguese (Brazil),^[25]^ obtaining a Cronbach’s alpha of 0.92 (95% CI: 0.90–0.93) and showing adequate internal consistency. The FSFI is a self-report questionnaire composed of 19 questions to evaluate female sexual functioning, which uses a Likert scale ranging from 0 to 5 and has 6 domains: desire, subjective arousal, lubrication, orgasm, satisfaction and pain (dyspareunia).^[26,27]^

Sexual function will be analyzed at baseline (*T*0), 6 weeks after the intervention (*T*1), and 12 weeks after the intervention (*T*2). In other studies that performed an intervention to improve the sexual functioning of women with breast cancer, the outcome was evaluated between 4 weeks and 12 months after the intervention.^[8,11]^

### 2.10. Blinding

Due to the nature of the intervention, it is impossible to blind the participants and the researcher responsible for providing and monitoring the intervention. Therefore, only research assistants who will evaluate the outcome after the intervention and the statistician who will perform the data analysis will be blinded concerning the participants’ group allocation.^[14]^

### 2.11. Strategies to improve adherence to interventions

Short Message Notifications will be sent to remember the subjects of the date and time of the scheduled sessions as a strategy to increase participants’ adherence to the study. Adherence will be assessed by participation in the telephone counseling sessions, which will be recorded in a specific form, including information such as the date and time of the intervention, duration, the topic addressed, and other relevant notes. In addition, a postintervention evaluation will include questions about the frequency, duration, and content offered through the counseling sessions.

### 2.12. Provisions for posttrial care

Adverse events will be evaluated by the number of participants who report possible worsening symptoms in the primary endpoint. In addition, participants will indicate whether they have experienced worsening symptoms of any kind in the postintervention survey.

### 2.13. Statistical methods

The data will be compiled and analyzed in the JASP program (Copyright 2013–2021 University of Amsterdam) Version JASP 0.15.^[27]^

Descriptive statistics will be obtained for the independent variables (means for continuous variables, absolute and relative frequencies for categorical variables, and their respective confidence intervals). An ANOVA statistical test for repeated measures will be completed to assess the extent to which the control and intervention groups differ concerning the outcome variable (sexual functioning) in the periods analyzed (*T*0, *T*1, and *T*2). All statistical analyses will be performed in collaboration with external statisticians, who will not be informed about the allocation of the groups.

The dataset generated or analyzed during this study will not be publicly available due to the ethics review act. However, it will be available from the corresponding author upon reasonable request and under the Brazilian guidelines for research collaboration and data transfer.

The study results will be submitted for publication in a peer-reviewed nursing journal. No professional writer will be involved.

### 2.14. Ethics approval and consent to participate

Ethical approval was obtained by the Research Ethics Committee of the Federal University of Ceará, Brazil, under opinion no. 461609 and CAAE no. 43072721.9.0000.5054; and the Maternity School Assis Chateaubriand, Ceará, Brazil, with opinion number 4742687 and certificate of ethical appreciation number 43072721.9.3002.5050.

Written informed consent will be obtained from all participants. All participants will receive a unique code number. The code will be stored separately from the survey data and will only be accessible to members of the research team. All data will be stored and processed under Brazilian law no. 13.709/2018 on data protection.^[29]^ The (Brazilian Clinical Trials Registry in Portuguese) will be notified of any changes in the trial registry if important changes regarding the conduction or protocol of the RCT are needed.

## 3. Results

Differences in levels of sexual function among women allocated to the control and intervention groups in the analyzed periods (*T*0, *T*1 and *T*2). In addition, there were differences in the scores of the domains of sexual function (desire, excitement, orgasm, satisfaction and pain).

## 4. Discussion

Some studies have already been developed using telehealth as a tool to apply educational interventions for breast cancer patients. However, different outcome variables have been studied, such as psychological suffering and coping,^[30]^ depression, fatigue, quality of life,^[24]^ and chemotherapy-related symptoms.^[31]^ A telephone counseling program assessed the improvement in psychosocial outcomes posttreatment of women with breast cancer to improve sexual health through variables such as anguish, depression, sexual dysfunction, and personal growth.^[10]^ Another study used a couple-based telephone intervention to address sexual issues.^[8]^ Furthermore, researchers have developed a multimodal nursing care program based on WeChat, consisting of chat software for the early rehabilitation of women after breast cancer surgery.^[32]^ However, none of these studies have specifically evaluated telenursing. The current study protocol describes the procedures for the clinical trial of telephone counseling.

Information and communication technologies and follow-up and monitoring systems, with an emphasis on telenursing, improve the health and self-care outcomes of people with one or more Chronic Noncommunicable Diseases. In addition, telenursing is considered a resource to improve the quality of life of these patients and reduce hospitalizations and costs. This tool has been used to educate patients and health professionals.^[33,34]^

The clinical trial design has several significant strengths. The first is that the RCT is considered the gold standard of clinical research, allowing more reliable conclusions regarding the effectiveness of interventions. In addition, the study has 2 parallel arms for comparing the intervention to usual care and will include a follow-up assessment of the outcome 12 weeks after the intervention in addition to the measurement that will take place immediately after the end of the intervention (6 weeks). Finally, the quality of the research data will possibly be high due to the use of validated instruments (such as the FSFI) for measuring sexual function. Regarding the weaknesses of the study, we can mention the nonmeasurement of the prevalence rate of sexual problems in the population and the uncertainty regarding the inclusion and retention rate, which can impact the time needed to achieve the sample size required for adequate statistical significance. Another limitation is the use of a nonactive treatment in the control group, which can generate a risk of general and nonspecific effects.

## 5. Conclusion

This evidence-based nursing care strategy can be used to improve the sexual function of breast cancer patients and consequently their quality of life and marital relationship.

## Authors contributions

**Data curation:** Iarlla Silva Ferreira.

**Investigation:** Romel Jonathan Velasco Yanez.

**Project administration:** Iarlla Silva Ferreira.

**Resources:** Ana Fátima Carvalho Fernandes.

**Software:** Romel Jonathan Velasco Yanez.

**Writing** – **original draft:** Iarlla Silva Ferreira and Ana Fátima Carvalho Fernandes.

**Writing** – **review & editing**: Régia Christina Moura Barbosa Castro and Andrea Rodrigues Bezerra.
